# Evaluation of Cover Crops and Biopesticides to Manage *Meloidogyne incognita* on Sweetpotatoes in Greenhouse and Microplot Settings

**DOI:** 10.2478/jofnem-2025-0015

**Published:** 2025-04-16

**Authors:** Claire M. Schloemer, Scott H. Graham, Koon-Hui Wang, Brent S. Sipes, Kathy S. Lawrence

**Affiliations:** 559 Devall Dr. CASIC Building, Auburn Univ, AL 36849; ALFA Building, Auburn Univ, AL 36849; 3050 Maile Way, Gilmore Hall, University of Hawai’i at Manoa, HI 96822; 3190 Maile Way, St. John Hall, University of Hawai’i at Manoa, HI 96822

**Keywords:** Integrated Nematode Management, *Meloidogyne*, organic, root-knot nematode, MeloCon and Chitocide

## Abstract

Interest in organic production is growing, highlighting the need for effective organic integrated management practices for sweetpotatoes. This study aimed to evaluate biopesticides and cover crops for managing *Meloidogyne incognita* in greenhouse and microplot settings. In the greenhouse, *M. incognita* reproduction factors were highest following field pea at 15.3 and crimson clover at 5.0, while daikon radish, elbon rye, and cover crop mixes had the lowest factors near 1.0. Summer cover crops sunn hemp, velvetbean, and ‘Piper’ sudangrass did not support *M. incognita* populations, with reproduction factors below 1. Greenhouse tests revealed similar *M. incognita* egg numbers/gram of root across all biopesticides, with MeloCon maintaining the lowest numbers. Microplot testing of the biopesticides on sweetpotatoes found *M. incognita* populations were similar to MeloCon, BotaniGard 22 WP plus Triple Threat Entomopathogenic Nematodes, Chitocide, Seduce, Promax, and Minuet. The highest marketable yield of 0.56 kg/plant was recorded in microplots treated twice with Chitocide, followed by BotaniGard 22 WP plus Triple Threat Entomopathogenic Nematodes, AzaGuard, and Majestene, all of which were comparable to synthetic nematicide, Velum applied at planting. In two field microplot trials, winter cover crops, black oat, daikon radish, and cover crop mixes of all cover crops tested produced the highest sweetpotato yield. Daikon radish, elbon rye, crimson clover, cover crop mix, black oats, and yellow mustard supported lower nematode populations compared to field peas. Overall, all cover crops tested, except field peas and crimson clover, reduced the *M. incognita* populations during the cover cropping season. Biopesticide MeloCon was most effective in reducing *M. incognita* populations post sweetpotato planting.

Sweetpotato (*Ipomoea batatas*) is a globally significant crop, ranking as the seventh most important food crop worldwide (FAO, 2023). Cultivated primarily for its starchy, nutrient-dense roots, sweetpotatoes are used for various purposes, including human consumption, animal feed, and industrial applications such as ethanol and biofuel production (Bovell-Benjamin, 2010). In the United States, the Southeast is the major sweetpotato-producing region, with the primary production states being Alabama, Louisiana, Mississippi, and North Carolina (USDA, 2023). The vegetable’s adaptability to tropical and subtropical regions, coupled with its drought tolerance and ability to thrive in low-fertility soils, makes sweetpotato well-suited for low-input production and makes it feasible to be produced organically (Mukhopadhyay, et al., 2011). Organic agriculture has increased in popularity, and there has been a rise in consumer demand for organic products, including fruits and vegetables (Lotter, 2003). Vegetables, which include sweetpotatoes, are cultivated on 57% of American organic farms (Greene and Kremen, 2003). Organic farming relies on ecologically based pest and fertility management strategies and avoids synthetic pesticides (Greene and Kremen, 2003). The perceived benefits of the organic production model include improved soil health, reduced pesticide usage, ecological harmony, and lower energy input (Lotter, 2003).

Plant-parasitic nematodes and insect pests pose a significant threat to sweetpotato crops, and in the Southeast, the southern root-knot nematode (*Meloidogyne incognita* (Kofoid and White)) is a particularly damaging species (Kim and Yang, 2019). *Meloidogyne incognita* infections result in root galling, reduced plant vigor, and lower yields (Bird, 1974). In conventional production, both fumigant and nonfumigant chemical nematicides are utilized, but these practices are not aligned with organic farming (Liu and Grabau, 2022). Consequently, organic growers turn to biopesticides, such as Majestene and MeloCon WG, and cultural practices, like winter cover cropping, to manage nematode infestations (Greene and Kremen, 2003; Timper et al., 2006).

Cover crops are defined by the Sustainable Agriculture Research and Education program as “a plant that is used primarily to slow erosion, improve soil health, enhance water availability, smother weeds, help control pests and diseases, increase biodiversity, and bring a host of other benefits to your farm” (Clark, 2015). Grasses and legumes are both grown as cover crops, but they affect the system differently. Legumes can reduce reliance on nitrogen fertilizers through nitrogen fixation, while grasses are effective in preventing erosion and nitrate leaching (Fageria et al., 2005). In the Southeastern United States, winter cover crops are established after harvest of the cash crop and terminated in spring by mowing, rolling/crimping, or herbicides, while summer cover crops are cultivated during the growing season as an alternative to the cash crop (Timper et al., 2021). Cover crops can also suppress plant-parasitic nematodes by acting as a non-host, producing allelopathic compounds, or supporting nematode antagonists (Wang et al., 2006). For example, rye (*Secale cereale* L.) and mustard (*Brassica nigra* L.) suppress plant-parasitic nematode populations through the production of toxic metabolites (Timper et al., 2021, Meyer et al., 2011). However, winter cover crops can pose the risk of the “green bridge effect”, by sustaining soil insect pests through winter, potentially increasing pest damage to subsequent cash crops, like sweetpotatoes (Pellegrino et al., 2021, Favetti et al., 2017). Thus, it is essential to carefully select and evaluate cover crops to optimize benefits while mitigating potential risks.

Sweet potato growers have various biological nematicides available to manage nematode infestations sustainably; however, products tend to fluctuate in market availability. In the U.S., these nematicides include Majestene (containing heat-killed *Burkholderia rinojensis* strain A396 cells and spent fermentation media) currently by ProFarm Group, and MeloCon WG (containing *Purpureocillium lilacinum* strain 251) by Certis Biologicals (Grabau and Noling 2021). Other biological control products beneficial to sweetpotato growers include Serenade (*Bacillus subtilis*) by Bayer Crop Science, which helps control root-knot and reniform nematodes by colonizing the roots and producing compounds that inhibit nematode development (Castillo et al., 2013). More biologicals are available on the market but not marketed for sweetpotatoes. There is a lack of a comprehensive and updated comparison of cover crops and biopesticides for sweet potato production. The objectives of this work were to evaluate multiple biopesticides as well as winter and summer cover crops for the management of *M. incognita* in the greenhouse and microplot setting to select the most effective treatments to be integrated for sweetpotato nematode management in the field.

## Materials and Methods

### Nematode inoculum

*Meloidogyne incognita* race 3 used for inoculum in these experiments was cultured on corn maintained in 500 cm^3^ polystyrene pots in the greenhouse at the Plant Science Research Center in Auburn, AL. All soil used to increase nematodes and in the greenhouse trials was a Kalmia loamy sand textured soil (80% sand, 10% silt, 10% clay, 1.2% organic matter, pH 6.9) sourced from Auburn University’s Plant Breeding Unit (Tallassee, AL). The soil was pasteurized at 88°C for 12 hours, allowed to cool for 24 hours and pasteurization was repeated. The pasteurized soil was combined with sand at a rate of 1:2 soil to sand. Fertilizer and lime were added at rates recommended by Auburn University Soil Testing Laboratory. Nematode eggs were extracted from the roots of approximately 60-days-old corn stock cultures. Corn shoots were discarded, and the roots were gently washed in water to remove excess soil. Nematode eggs were extracted through a modified method by Hussey and Barker by placing the roots in a 0.625% NaOCl solution and shaking at 1 G force for four minutes on a Barnsted Lab Line Max Q 5000E Class shaker (Thermo Fisher Scientific: Waltham, MA) (Hussey and Barker, 1973). The roots were gently scrubbed under running water, and dislodged eggs were collected on a 25 µm pore sieve. Using a modified centrifugation method, the egg solution was transferred into 50 mL centrifuge tubes, processed by sucrose centrifugation-flotation in 1.14 specific gravity, and centrifuged at 1400 rpm for one minute (Jenkins 1964),. The eggs in the supernatant of the sucrose solution were collected on a 25 µm pore sieve and rinsed well with water. *Meloidogyne incognita* egg density was determined by enumerating the eggs using a Nikon TSX 100 inverted microscope at 40X magnification. Egg density was adjusted to 5,000 eggs/mL.

### Greenhouse tests

#### Biopesticides

Trials to evaluate the impacts of biopesticides on *M. incognita* race 3 were conducted initially in the greenhouse. Ten biopesticides AzaGuard (a.i. Azadirachtin, BioSafe Systems, LLC, Hartford, CT), BotaniGard 22 WP (a.i. *Beauveria bassiana* strain GHA, Certis Biologicals, Columbia, MD), BoteGHA ES (a.i. *Beauveria bassiana* strain GHA, LAM International Corporation, Butte, MT), Chitocide (a.i. Quillaja extract and Chitosan, Concept AgriTek, Charleston, MO), Majestene (a.i. heat-killed *Burkholderia* spp. strain A396 cells and spent fermentation media, ProFarm Group, Davis, CA), MeloCon (a.i. *Paecilomyces lilacinus* strain 251, Certis Biologicals, Columbia, MD), Minuet (a.i. *Bacillus subtilis* strain QST 713, Bayer CropScience, St. Louis, MO), Monterey Nematode Control (a.i. Saponins of *Quillaja saponaria*, Lawn and Garden Products, Inc., Fresno, CA), Promax (a.i. thyme oil, Bio Huma Netics, Inc., Gilbert, AZ), and Seduce (a.i. Spinosad, Certis Biologicals, Columbia, MD), plus the conventional product Velum Prime (a.i. fluopyram, Bayer CropScience, St. Louis, MO) were compared to an untreated control. One 15 cm ‘Beauregard’ variety sweetpotato slip was planted per 500 cm^3^ polystyrene cup (Dart Container Corporation, Mason, Michigan) filled with the pasteurized soil mixture. At planting, 5,000 *M. incognita* eggs were pipetted to each pot in a 2.5-cm depression in 1 mL of water and covered with soil to prevent desiccation. After nematode inoculation, the biopesticide treatments were applied at labeled rates (Table 1) via 50 mL drench treatments. The biopesticide treatments were applied 30 days after planting for the second application.

**Table 1:** Effect of biopesticides on sweetpotato plant growth parameters and *Meloidogyne incognita* race 3 nematode reproduction when grown in the greenhouse.

**Biological control**			**Root fresh weight (g)**	**Shoot fresh weight (g)**	**Biomass^a^ (g)**	***M incognita* eggs/root system**	***M. incognita* eggs/g root^b^**
Name	Active ingredient	Rate					
Untreated	None	None	36 a^c^	35 a	71 a	114,778 ab	3,607 ab
AzaGuard	Azadirachtin	1.1 L/ha	37 a	32 a	69 a	83,183 ab	2,316 ab
BotaniGard 22 WP + Triple Threat Entomopathogenic Nematodes	*Beauveria bassiana + Steinernema feltiae, Steinernema carpocapsae, Heterorhabditis bacteriophora*	2.3 L/ha	39 a	31 a	70 a	151,634 a	5,180 a
BoteGHA ES	*Beauveria bassiana*	4.9 kg/ha + 123.5 million IJ’s/ha	42 a	37 a	80 a	121,832 ab	3,039 ab
Chitocide	Quillaja extract and chitosan	1.2 kg/ha	43 a	31 a	74 a	97,874 ab	2,817 ab
Majestene	Heat-killed *Burkholderia spp.*	18.7 L/ha	37 a	35 a	76 a	129,140 ab	4,657 a
MeloCon	*Purpureocillium lilacinum*	0.7 L/ha	40 a	35 a	75 a	73,018 ab	2,086 ab
Minuet	*Bacillus subtilis*	1.5 L/ha	39 a	33 a	72 a	96,509 ab	2,813 ab
Monterey Nematode Control	Saponins of *Quillaja saponaria*	2.5 L/ha	37 a	35 a	73 a	132,973 ab	4,189 ab
Promax	Thyme oil	12.4 L/ha	42 a	35 a	77 a	134,160 a	3,929 ab
Seduce	Spinosad	33.6 kg/ha	49 a	34 a	84 a	117,961 ab	2,806 ab
Velum	Fluopyram	0.4 L/ha	43 a	33 a	72 a	7,563 b	194 b

*P* value^d^			0.2123	0.9370	0.7363	0.0239^**^	0.0375^**^

a Biomass is the sum of root and shoot fresh weight in grams.

b *Meloidogyne incognita* eggs/g of fresh root weight.

c Values followed by the same letter are not significantly different at *P* ≤ 0.05 as determined by the Tukey Kramer Method.

d *P*-values for Type III fixed effects with significance at the 0.1, 0.05, 0.01, and 0.001 level is indicated by ^*^, ^**^, ^***^, and ^****^respectively.

#### Winter and summer cover crops

To determine the reproduction rate of *M. incognita* race 3 on winter cover crops, seven individual winter cover crops and two winter cover crop mixes were selected. Individual winter cover crops tested included black oats (*Avena strigose*), crimson clover (*Trifolium incarnatum*), daikon radish (*Raphanus sativus* var. longipinnatus), elbon rye (*Secale cereale*), field pea (*Pisum sativum*), wheat (*Triticum aestivum*), and yellow mustard (*Sinapis alba*). Winter cover crop mix 1 consisted of crimson clover, field peas, yellow mustard, black oat, daikon radish, and elbon rye, while winter cover crop mix 2 consisted of crimson clover, daikon radish, elbon rye, and wheat. Summer cover crops included Peper sudangrass (*Sorghum bicolor*), Sunn hemp (*Crotalaria juncea*), and Velvetbean (*Abutilon theophrasti*). All cover crops were compared to a fallow unplanted control. Seeds were obtained from Piedmont Fertilizer Company (Opelika, AL) and planted at recommended rates into 500-cm^3^ polystyrene cups. Reducing the recommended rate for planting in the field to pots in the greenhouse left two to three seeds per pot. The mixes were planted with one seed of each cover crop to make sure all were represented. The cover crops test was inoculated with 5,000 *M. incognita* eggs pipetted to each pot two weeks after cover crop planting when the cover crop seedlings had emerged.

#### Experimental design

The biopesticides and winter cover crops in each set of tests were arranged in a randomized complete block design (RCBD) with five replications, and each experiment was repeated. Plants were watered as needed to maintain soil moisture. Greenhouse temperatures ranged from 25°C to 29°C over the course of this test. Lighting was provided by 1000-watt halide bulbs which produced 110,000 lumens for a 14-hour day length.

Entire sweetpotato plants were collected, and plant measurements were taken from the biopesticide tests approximately 45 days after planting (DAP) and from the cover crop tests at 74 DAP. Plant measurements included root fresh weight (RFW), shoot fresh weight (SFW), and biomass (RFW + SFW). *Meloidogyne incognita* population density was also recorded by extracting the total number of eggs from each root system and was reported as eggs per gram of root. Nematodes were extracted from the roots using a combination of gravity sieving and sucrose centrifugal flotation (previously described) and enumerated with a Nikon TSX 100 inverted microscope at 40× magnification.

### Microplot tests

Biopesticide effects on *M. incognita* race 3 were again tested in microplate experiments established at the Plant Science Research Center (PSRC) in Auburn, AL. Summer cover crops were not included in microplot experiments. Each microplot consisted of a pot within a pot design with a 23-L plastic tree pot (Grip Lip 2800; Nursery Supplies Inc., Montgomery, AL) nested inside an identical 23-L plastic pot with a brick placed in between to serve as a root barrier and then buried into the ground. Microplots were filled with a Kalmia loamy soil (fine-loamy over sandy or sandy skeletal, siliceous, semiactive, thermic Typic Hapludults) comprised of 80% sand, 10% silt, and 10% clay mixed as 2 parts field soil with 1 part sand. Water was provided through a drip irrigation system and was adjusted throughout the season as needed. Each microplot was inoculated with 250 cm^3^ of soil which contained an average of 50,000 eggs and J2 life stages of *M. incognita* race 3. The biopesticides test took place from June to October 2022. Biopesticides tested were identical to those used in the greenhouse test. For this test, one 15 cm ‘Beauregard’ variety sweetpotato slip was planted per microplot on 3 June 2022, and all nematicide treatments were applied at labeled rates via a 0.5 L drench treatment at planting and again at 35 DAP (8 July 2022). Treatments were arranged in a RCBD with 5 replications and the test was repeated once. Soil samples were taken at 103 DAP (14 September 2022) to determine the efficacy of biopesticides on soil populations of *M. incognita*. The sampling method consisted of collecting four 2.5-cm × 20-cm soil cores at the base of the plant from each microplot, composited prior to taking a 100 cm^3^ subsample for nematode extraction. Each soil sample was placed in a bucket with approximately 1 liter of water, where it was then swirled thoroughly to suspend the soil in the water. The mixture was then poured through nested 250 µm-pore and 25 µm-pore sieves, and the contents left on the 25 µm-pore sieves were collected and washed into 50 mL centrifuge tubes (Riggs and Schmitt, 1988). This process was followed by the sucrose centrifugal floatation method (Jenkin, 1964). *Meloidogyne incognita* J2 population levels were determined by enumerating the nematodes extracted from the soil using a Nikon TSX 100 inverted microscope at 40× magnification. At plant maturity, near 130 DAP, microplots were harvested by removing all marketable sweetpotato roots per microplot and their weights were recorded.

#### Winter cover crop tests

Two winter cover crop microplot tests were planted concurrently with treatments arranged in a RCBD with 5 replications on 18 November 2022. Winter cover crops selected for the microplot test included black oats, crimson clover, daikon radish, elbon rye, field pea, and yellow mustard. One winter cover crop mixture was included and consisted of crimson clover, field pea, yellow mustard, black oat, daikon radish, and elbon rye. The recommended seeding rate for planting in the field was adjusted to one square foot or 0.1 square meters for the microplot. The mixes were planted with at least two seeds of each cover crop to make sure all were represented. Winter cover crops grew throughout winter and were terminated by removing the aboveground biomass and incorporating the roots into the soil in early May 2023. One 15 cm ‘Beauregard’ variety sweetpotato slip was planted per microplot for the first test on 26 May 2023 and for the second test on 2 June 2023. Soil samples were taken on 18 July 2023 and 25 July 2023 (53 DAP) to determine the effect of winter cover crops on soil populations of *M. incognita*. Soil samples were extracted to measure nematode populations and enumerated as previously described. Microplots were harvested at plant maturity on 19 September 2023 and 26 September 2023 (116 DAP).

### Statistical analysis

Data collected from the winter cover crop and biopesticide greenhouse and microplot trials were analyzed by SAS 9.4 (SAS Institute: Cary, NC) using the PROC GLIMMIX procedure. The analysis of variance was conducted with biopesticides or cover crops as the main factor. Student panels were produced to determine the normality of the residuals. The tests also found no significant interactions between trial and treatment for the greenhouse and microplot tests, and thus, data from the repeated trials for each experiment were combined for analysis. LS-means were compared between the cover crop treatments and biopesticides by Tukey Kramer LS-means test at *P* ≥ 0.05. LS-means presented in the tables followed by different letters indicate a significant difference.

## Results

### Greenhouse testing

#### Biopesticide Experiments

*Meloidogyne incognita* race 3 nematode population density increased to high levels when grown on sweetpotato over the 45-days greenhouse test (Table 1). Biopesticides did not significantly affect sweetpotato root fresh weight, shoot fresh weight, or total biomass, with all biopesticides producing similar plant weights. No phytotoxicity was observed on the sweetpotatoes with any of the products applied. Velum was the most efficacious product tested and reduced *M. incognita* eggs per root system by 93% compared to the untreated control. Of the biopesticides, Promax and the combination of BotaniGard 22 WP and Triple Threat Entomopathogenic Nematodes supported significantly (*P* ≥ 0.05) higher *M. incognita* eggs per root system than Velum but were not significantly different from the other biopesticides tested. All biopesticides supported similar populations of *M. incognita* eggs per root system and eggs per gram of root. MeloCon supported the lowest number of *M. incognita* eggs per gram of root among biopesticides tested. All the biopesticides maintained *M. incognita* population levels at <3,300 eggs per gram of root. Velum supported significantly (*P* ≥ 0.05) fewer *M. incognita* eggs per gram of root than the other biological control products tested and the untreated control (*P* ≤ 0.05).

#### Winter cover crops

**Figure 1:**
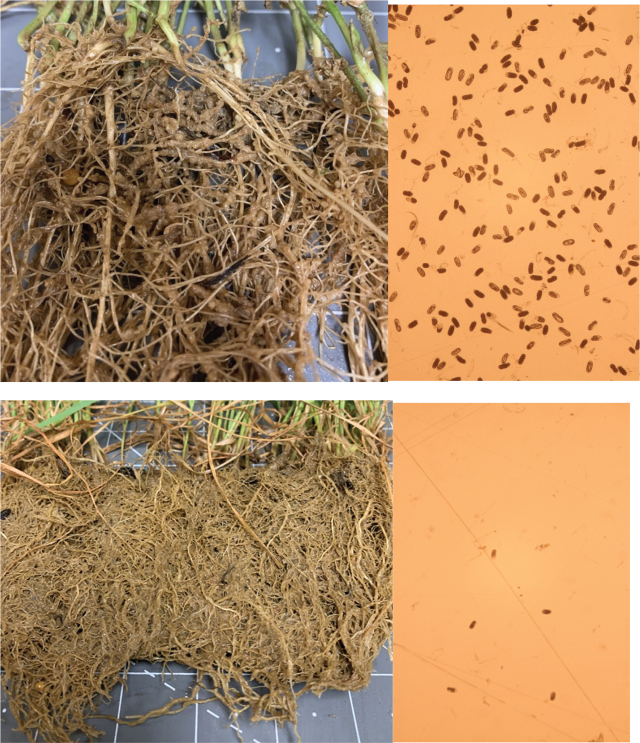
Field pea root system (top image) with symptomatic galling and the *Meloidogyne incognita* eggs extracted at 40 X. Below Elbon rye without galling and the eggs observed at 40X.

*Meloidogyne incognita* population densities increased to high levels on the winter cover crops in the greenhouse over the 56-days test (Table 2). The highest root fresh weight was recorded on cover crop mix 1 (crimson clover, field pea, yellow mustard, black oat, daikon radish, and elbon rye), which was numerically similar to cover crop mix 2 (crimson clover, daikon radish, elbon rye, and wheat), elbon rye, black oat, and wheat (*P* ≤ 0.05). Crimson clover, field pea, and yellow mustard had the lowest root fresh weights in this test and were not significantly different from the fallow (*P* ≤ 0.05). Shoot fresh weights were 79% lower than the root weights with daikon radish producing the largest plants (*P* ≤ 0.05) compared to all other winter cover crops. Biomass varied across winter cover crops, with the highest biomass (*P* ≤ 0.05) recorded on cover crop mix 1 and 2, elbon rye, black oats, and wheat (*P* ≤ 0.05). The majority of winter cover crops evaluated supported low *M. incognita* densities. Elbon rye supported the fewest *M. incognita* eggs per gram of root and was statistically similar to all other cover crops tested, excluding field peas (*P* ≤ 0.05). The highest *M. incognita* nematode population density was found on field peas, which supported a 98% greater *M. incognita* nematode population per gram of root (*P* ≤ 0.05) than the average of all the remaining cover crops (Figure 1). Crimson clover also supported a high population of *M. incognita* nematodes with 94% higher population density than the average of all the remaining cover crops; however, it was not significant at the *P* ≤ 0.05. Excluding fallow, the lowest *M. incognita* nematode reproductive factors of 1.0 were recorded on daikon radish and cover crop mix 2. This indicates that these winter cover crops were found to be poor winter hosts of *M. incognita* race 3 only allowing the nematode to sustain its population. Mix 1, elbon rye, yellow mustard, and black oats would also be considered poor hosts supporting minimal nematode reproduction. High reproductive factors of 15.3 and 5.0 were recorded on field peas and crimson clover, respectively. Those two cover crops could increase *M. incognita* populations before the summer crop planting.

**Table 2:** Effect of winter and summer cover crops on plant growth and *Meloidogyne incognita* race 3 reproduction in the greenhouse.

**Winter cover crop**	**Root fresh weight (g)**	**Shoot fresh weight (g)**	**Biomass^a^ (g)**	***Meloidogyne incognita* eggs/g root^b^**	**Reproductive factor^c^**
Fallow	0 c^d^	0 d	0 d	0 b	0.2
Black oats	141 ab	26 b	167 ab	105 b	1.6
Crimson clover	16 c	13 c	29 d	1,834 b	5.0
Daikon radish	76 bc	45 a	121 bc	79 b	1.0
Elbon rye	166 a	16 d	182 ab	48 b	1.1
Field peas	24 c	17 c	41 cd	5,430 a	15.3
Wheat	145 ab	18 c	162 ab	160 b	2.5
Yellow mustard	36 c	29 b	65 cd	352 b	1.5
Mix 1^e^	171 a	29 b	200 a	51 b	1.1
Mix 2^f^	170 a	21 bc	191 ab	62 b	1.0

*P* value^g^	0.0001^****^	0.0001^****^	0.0001^****^	0.0001^****^	

Summer cover crop					
Fallow	0 c	0 d	0 c	0 b	0.002
Elbon rye	182 ab	30 c	213 ab	288 a	0.058
Piper sudangrass	244 a	58 ab	303 a	176 ab	0.035
Sunn hemp	130 b	50 b	181 b	23 ab	0.005
Velvetbean	101 b	70 a	171 b	18 b	0.004

*P* value^g^	0.0001^****^	0.0001^****^	0.0001^****^	0.0136^**^	

a Biomass is the sum of root and shoot fresh weight in grams.

b *Meloidogyne incognita* eggs/g of fresh root weight.

c Calculated by dividing the final population of *M. incognita* by its initial population.

d Values followed by the same letter are not significantly different at *P* ≤ 0.05 as determined by the Tukey Kramer Method.

e Mix 1 contained crimson clover, field pea, yellow mustard, black oat, daikon radish, and elbon rye.

f Mix 2 contained crimson clover, daikon radish, elbon rye, and wheat.

g *P*-values for Type III fixed effects with significance at the 0.1, 0.05, 0.01, and 0.001 level is indicated by ^*^, ^**^, ^***^, and ^****^ respectively.

#### Summer cover crops

Of the summer cover crops, piper sudangrass and elbon rye produced the highest root fresh weight (*P* ≤ 0.05). The summer covers velvet bean, and piper sudangrass supported the largest shoot fresh weights (P ≤ 0.05). However, the shoot weights were 80% lower than the root fresh weights. Biomass (sum of root and shoot fresh weights), which was influenced by the root weights, was greatest (P ≤ 0.05) for piper sudangrass and elbon rye, with elbon rye supporting similar biomass as sunn hemp and velvet bean (Table 2). *Meloidogyne incognita* race 3 reproduction was highest on elbon rye which supported 288 *M. incognita* eggs per gram of root at the conclusion of the test and a reproductive factor of 0.058. Sunn hemp and velvetbean both supported low levels of *M. incognita* reproduction with reproductive factors of 0.005 and 0.004, respectively. Velvet bean supported the lowest *M. incognita* populations of the summer cover crops with only 18 *M. incognita* eggs per gram of root, a 94% decrease (*P* ≤ 0.05), compared to elbon rye.

### Microplot testing

#### Biopesticides

All biopesticides tested reduced *M. incognita* population densities on sweetpotatoes below the untreated control in these microplot conditions (Table 3). The lowest *M. incognita* population density of 765 and 773 J2 nematodes/100cm^3^ soil was recorded in the microplots treated with MeloCon (*P* ≤ 0.05), and the combination of BotaniGard 22 WP and Triple Threat Entomopathogenic Nematodes, respectively. Ranking the biopesticides found *M. incognita* populations were lowest (*P* ≤ 0.0001) when sweetpotatoes were treated with MeloCon, the combination of BotaniGard 22 WP, and Triple Threat Entomopathogenic Nematodes, Chitocide, Seduce, Promax, and Minuet. These biopesticides maintained *M. incognita* population levels at less than 900 J2/100cm^3^ soil. Biopesticide treatments had no significant effect on marketable yield in the microplots. Although not statistically significant, ranking the biopesticides for yield found the highest marketable yield of 0.56 kg/plant was recorded on the plants treated with Chitocide, followed by BotaniGard 22 WP, and Triple Threat Entomopathogenic Nematodes, Velum, AzaGuard, and Majestene.

**Table 3:** Effect of biopesticides on *Meloidogyne incognita* race 3 reproduction and sweetpotato yield in a microplot setting.

	**Biopesticide**		***M. incognita*/100 cm^3^ soil**	**Marketable yield/plant (kg)**
Name	Active ingredient	Rate		
Untreated	None	None	1,236 a^a^	0.509 a
AzaGuard	Azadirachtin	1.1 L/ha	1,082 c	0.513 a
BoteGHA ES	*Beauveria bassiana*	2.3 L/ha	1,190 ab	0.442 a
BotaniGard 22WP + Triple Threat Entomopathogenic Nematodes	*Beauveria bassiana + Steinernema feltiae, Steinernema carpocapsae, Heterorhabditis bacteriophora*	4.9 kg/ha + 123.5 million IJ’s/ha	773 f	0.533 a
Chitocide	Quillaja extract and chitosan	1.2 kg/ha	819 e	0.558 a
Majestene	Heat-killed *Burkholderia spp.*	18.7 L/ha	1,043 c	0.505 a
MeloCon	*Purpureocillium lilacinum*	0.7 L/ha	765 f	0.378 a
Minuet	*Bacillus subtilis*	1.5 L/ha	881 d	0.460 a
Monterey Nematode Control	Saponins of *Quillaja saponaria*	2.5 L/ha	920 d	0.367 a
Promax	Thyme oil	12.4 L/ha	834 e	0.432 a
Seduce	Spinosad	33.6 kg/ha	819 e	0.418 a
Velum	Fluopyram	0.4 L/ha	1,159 b	0.517 a

*P* value^b^			0.0001^****^	0.9743

a Values followed by the same letter are not significantly different at *P* ≤ 0.05 as determined by the Tukey Kramer Method.

b *P*-values for Type III fixed effects with significance at the 0.1, 0.05, 0.01, and 0.001 levels are indicated by ^*^, ^**^, ^***^, and ^****^, respectively.

#### Winter cover crops

Although more summer cover crops are effective in suppressing *M. incognita*, winter cover cropping is a preferred crop rotation practice in the Southern U.S. Of the winter cover crops tested in the microplots, field peas, elbon rye, and mix 1 (crimson clover, daikon radish, elbon rye, and wheat) produced the highest (*P* ≤ 0.05) aboveground biomass at cover crop termination (Table 4). Yellow mustard and the weedy fallow produced the lowest biomass, which was not significantly different from black oats and crimson clover. Prior to sweetpotato planting, field peas supported the highest soil population of *M. incognita* (35 J2/100cm^3^ soil), followed by fallow. Daikon radish, elbon rye, crimson clover, cover crop mix 1, black oats, and yellow mustard all supported lower nematode populations than the field peas. At the July sampling date (60 DAP), when sweetpotatoes were growing following the cover crops, the *M. incognita* population density was statistically equivalent across all covers. This trend continued at the September sampling date, with no difference in *M. incognita* populations on the sweetpotatoes following the winter cover crops. Sweetpotato yields were similar across the winter cover crops as well. Ranking numerically, the highest sweetpotato yields were harvested from the plots that had been cultivated with black oat, daikon radish, and elbon rye.

**Table 4:** *Meloidogyne incognita* race 3 reproduction and sweetpotato yield following winter cover crops in a microplot setting.

**Winter cover crop**	**Winter cover crop biomass^a^ (g)**	**April *M. incognita*/100cm^3^ soil**	**July *M. incognita*/100cm^3^ soil**	**September *M. incognita*/100cm^3^ soil**	**Sweetpotato yield (kg/plant)**
Fallow	18 d^b^	30 ab	421 a	470 a	0.90 a
Black oats	51 bcd	22 dc	305 a	333 a	1.43 a
Crimson clover	44 bcd	19 dc	570 a	407 a	0.92 a
Daikon radish	32 dc	17 d	282 a	437 a	1.36 a
Elbon rye	70 abc	18 d	330 a	317 a	1.30 a
Field peas	97 a	35 a	416 a	385 a	1.05 a
Yellow mustard	27 d	26 bc	879 a	507 a	1.10 a
Mix 1^c^	84 ab	21 dc	393 a	317 a	1.38 a

*P* value^d^	0.0001^****^	0.0001^****^	0.0661^*^	0.0001^****^	0.2458

a Winter cover crop biomass was assessed as dry weight of aboveground biomass.

b Values followed by the same letter are not significantly different at *P* ≤ 0.1 as determined by the Tukey Kramer Method.

c Mix 1 contained crimson clover, daikon radish, elbon rye, and wheat.

d *P*-values for Type III fixed effects with significance at the 0.1, 0.05, 0.01, and 0.001 level is indicated by ^*^, ^**^, ^***^, and ^****^ respectively.

## Discussion

### Greenhouse testing

MeloCon was the most efficacious biopesticide, as it supported numerically the lowest populations of *M. incognita* compared to the other bio-based products in greenhouse testing. Similar results were found by Baidoo et al. (2017) on an ornamental shrub where an at-plant treatment of MeloCon resulted in significantly lower *M. incognita* per gram of root than the untreated control. However, they found that MeloCon did not result in higher cut foliage yield or plant growth. We also found that MeloCon did not significantly improve sweetpotato plant biomass, most likely due to ideal growing conditions despite nematode infection.

Velum, the chemical nematicide, outperformed all biopesticides tested in the greenhouse, resulting in the lowest *M. incognita* per gram of root. Chemical nematicides often outperform biopesticides when compared, and this finding is similar to that of Xiang et al. (2017), who found that all plant-growth-promoting rhizobacteria strains tested caused lower *M. incognita* mortality when compared with chemical controls aldicarb or abamectin (Hussain et al., 2017).

In the winter cover crop greenhouse test, field peas supported significantly higher *M. incognita* per gram of root than all other winter cover crops tested. This indicates that field peas are a good host of *M. incognita*, with a reproductive factor of 15.3 after 8 weeks of plant growth. Other research corroborates field pea (*Pisum sativum*) to be an important host of several species of root-knot nematode, including *M. incognita* (Haidar et al., 2009). Crimson clover also supported elevated *M. incognita* populations in the greenhouse, which agrees with findings from Timper et al. (2006) that crimson clover was an excellent host for *M. incognita* under greenhouse conditions. Therefore, field pea and crimson clover should not be planted alone as a winter cover crop prior to sweetpotato planting in fields infested with M. *incognita.* Elbon rye supported the lowest *M. incognita* population per gram of root in the greenhouse, which reinforces previous research that rye is a relatively poor host of *M. incognita* (Timper et al., 2006). The cover crop mixtures examined in these trials supported very low reproductive factors even though they contained the two legumes (crimson clover and field pea) that supported high nematode reproduction. The combination of the grasses and legumes appears to have allowed or enhanced the benefits of each cover crop similar to that suggested by Chapagain et al. (2020).

When testing summer cover crops, McSorley (1999) found that certain legumes like sunn hemp and velvetbean are very desirable because they are highly resistant to *Meloidogyne* spp. and are helpful for nitrogen management. Our greenhouse experiment reinforced this finding where we found the lowest *M. incognita* populations on sunn hemp and velvet bean, numerically. Wang et al. (2004) also emphasized the utility of sunn hemp as a plant-parasitic nematode suppressant cover crop when tested against *Meloidogyne spp.* and *Rotylenchulus reniformis.* They found that sunn hemp suppressed plant-parasitic nematode populations for 2 months after cash crop planting, resulting in significantly lower cash crop root gall ratings than the bare ground treatment (Wang et al., 2011). Velvetbean has also been shown to be effective in plant-parasitic nematode management. Weaver, Kabana, and Carden (1998) found that summer cover cropping with velvet bean reduced both Meloidogyne spp. and Heterodera glycine populations to undetectable levels by fall in both locations tested. Additionally, the low vining growth habit of velvet bean can aid in weed suppression (Weaver et al., 1998).

### Microplot testing

Although Velum performed the best in suppressing *M. incognita* race 3 under greenhouse conditions, this was not the case in the microplots evaluations where Velum supported the highest soil *M. incognita* population of all products tested, second only to the untreated control. This is likely due to later sampling timing in the microplots. Colyer et al. (1997) found that the effect of non-fumigant nematicides is typically restricted to the first few weeks after planting, and soil samples were taken in September, three months after Velum was applied. Fluopyram, the active ingredient in Velum, has been reported to have a half-life of 64.2 days in soil, which suggests that the sampling timing at sweetpotato harvest (103 DAP) did not capture the full potential of its nematicidal activity (Zheng et al., 2014). Further, similar end-of-the-season high nematode populations have been reported with *M. incognita* and R. reniformis when aldicarb was applied at crop planting (Lawrence and McLean, 2002; Jones et al., 2006). Thus, organic biopesticides applied regularly could outperform synthetic nematicides for a long-term crop like sweetpotato.

The findings from the microplot winter cover crop experiment confirmed that field peas were the most susceptible host to *M. incognita* out of all other winter cover crops tested here and should not be planted in fields infested with this root-knot nematode. However, when mixing field peas with the other cover crop (black oat, crimson clover, daikon radish, rye, wheat, yellow mustard) as Mix 1, it diluted the susceptibility of field peas to *M. incognita* in parts due to lower population densities of *M. incognita* supported by elbon rye and daikon radish (Table 4). Previous research has shown that the incorporation of a rye winter cover crop reduces soil populations of *M. incognita* J2’s (Johnson and Motsinger, 1989). This reduction has been attributed to the allelopathic compounds like benzoxazinoids from rye (Zasada et al., 2005; Zasada et al., 2007), or glucosinolate from daikon radish (Waisen et al., 2022), both of which generate degradation products toxic to *M. incognita.* However, the current study showed that the allelopathic effects of rye or daikon dissipated 2 months after sweetpotato was planted (July 2023 sampling).

#### Summary

Overall, our findings showed that some variability in *M. incognita* suppression was observed between the greenhouse and microplot tests. While none of the biopesticides reduced *M. incognita* egg counts in the greenhouse tests, MeloCon was the most promising biopesticide against *M. incognita*. Most interestingly, many biopesticides tested if applied twice during the sweetpotato crop were more suppressive to *M. incognita* compared to the one-time application of Velum. The winter grass cover crops elbon rye and daikon radish, along with their mixtures containing crimson clover, daikon radish, elbon rye, and wheat, resulted in lowered soil *M. incognita* populations when compared with leguminous winter cover crops field peas and crimson clover. Determining the most effective biopesticides and cover crops could aid decision-making to design an integrated nematode management approach to support sweetpotato growth and provide an economic return on yield in a *M. incognita* infested field.
